# Elevated Expression of ADAM10 in Skeletal Muscle of Patients with Idiopathic Inflammatory Myopathies Could Be Responsible for FNDC5/Irisin Unbalance

**DOI:** 10.3390/ijms24032469

**Published:** 2023-01-27

**Authors:** Roberta Zerlotin, Marco Fornaro, Mariella Errede, Patrizia Pignataro, Clelia Suriano, Maddalena Ruggieri, Silvia Colucci, Florenzo Iannone, Maria Grano, Graziana Colaianni

**Affiliations:** 1 Department of Precision and Regenerative Medicine and Ionian Area (DiMePRe-J), University of Bari Aldo Moro, Piazza Giulio Cesare 11, 70124 Bari, Italy; 2 Department of Translational Biomedicine and Neuroscience (DiBraiN), University of Bari Aldo Moro, Piazza Giulio Cesare 11, 70124 Bari, Italy

**Keywords:** irisin, FNDC5, ADAM10, myopathy, dermatomyositis, immune-mediated necrotizing myopathy

## Abstract

Dermatomyositis (DM) and immune-mediated necrotizing myopathy (IMNM) are two rare diseases belonging to the group of idiopathic inflammatory myopathies (IIM). Muscle involvement in DM is characterized by perifascicular atrophy and poor myofiber necrosis, while IMNM is characterized by myofiber necrosis with scarce inflammatory infiltrates. Muscle biopsies and laboratory tests are helpful in diagnosis, but currently, few biomarkers of disease activity and progression are available. In this context, we conducted a cohort study of forty-one DM and IMNM patients, aged 40–70 years. In comparison with control subjects, in the muscle biopsies of these patients, there was a lower expression of FNDC5, the precursor of irisin, a myokine playing a key role in musculoskeletal metabolism. Expectedly, the muscle cross-sectional areas of these patients were reduced, while, surprisingly, serum irisin levels were higher than in CTRL, as were mRNA levels of ADAM10, a metalloproteinase recently shown to be the cleavage agent for FNDC5. We hypothesize that elevated expression of ADAM10 in the skeletal muscle of DM and IMNM patients might be responsible for the discrepancy between irisin levels and FNDC5 expression. Future studies will be needed to understand the mechanisms underlying exacerbated FNDC5 cleavage and muscle irisin resistance in these inflammatory myopathies.

## 1. Introduction

Idiopathic inflammatory myopathies (IIM), also known as myositis, represent a group of autoimmune muscle diseases [1,2] usually occurring between 40 and 60 years of age or between 5 and 15 years of age in the juvenile form [3,4,5,6].

Symptomatology associated with IIM consists of muscular manifestations and often extramuscular manifestations, including myocarditis, skin rashes, and lung disease [7]. Muscular histology is characterized by inflammation, with lymphocytic and macrophage infiltration, loss of muscle fibers, and fibrosis [2,8]. Moreover, clinical and histological manifestations have been linked to the presence of specific autoantibodies, the so-called myositis-specific autoantibodies (MSA), found in 60% of patients with IIM. Based on clinical and pathological manifestations, IIM has been classified into four main forms: polymyositis (PM), dermatomyositis (DM), necrotizing myopathy (IMNM), and myositis with included bodies (IBM) [9,10,11,12,13].

To date, exercise has been shown to improve muscle function and quality of life in adult IIM patients [14,15,16]. Recent studies showed the positive effects of exercise are achieved both through the mechanical load applied to the musculoskeletal system and through the release of myokines during muscle contraction that act locally or systemically on different body districts [17,18]. Among them, the myokine irisin is well-known as an exercise mimetic hormone with pleiotropic effects. Irisin derives from the proteolytic cleavage of extracellular domain of its precursor, the fibronectin type III domain-containing protein 5 (FNDC5), expressed on the membrane of muscle fibers. The discovery of the autocrine anabolic effects of irisin on muscle paved the way for considering this molecule as a candidate for monitoring or potential treatment of muscle atrophy [17,18,19,20,21,22,23,24]. Six years after the discovery of irisin, the same authors identified its receptor that belongs to the αV class of integrins [25], and only very recently, it has been shown in cardiac muscle that FNDC5 is cleaved into irisin in an ADAM10-dependent manner [26]. ADAM10 belongs to the family of disintegrin and metalloproteinases (Adam), which are ubiquitous proteases with a common modular structure. They cross the membrane via transmembrane domain linking a short cytoplasmic C-terminal tail with complex extracellular domains consisting of disintegrin and metalloproteinase domains. Of note, the ADAM10 metalloproteinase has drawn considerable interest because it cleaves several protein substrates, thereby regulating turnovers of as many transmembrane proteins involved in cell adhesion and receptor signaling, through a process called ectodomain shedding [27]. Inflammatory myopathies have provided fertile ground for studying the involvement of these proteases in immune-mediated disease since proteolytic breakdown is crucial for immune cell infiltration and subsequent tissue destruction in muscle inflammatory disorders. A previous study showed some Adam proteins, expressed by specific tissues during the genesis of the immune-mediated pathology, correlate with the pathological conditions of IIM, and ADAM10 was localized exclusively in muscle fibers [28].

In this context, the present study was designed to explore in a cohort of IIM patients the expression of FNDC5 in muscle biopsies and serum concentrations of circulating irisin, showing their levels were unbalanced probably by increased ADAM10 expression.

## 2. Results

### 2.1. General Characteristics of Patients

Table 1 shows the demographic, anthropometric, skeletal muscle mass and laboratory parameters of the participants expressed as mean (±SD) or median (25%; 75%), as appropriate. This cohort study included forty control subjects (diagnosed as no-IIM) and forty-one patients with IIM of both sexes. Of these forty-one IIM patients, thirty-two (78%) were diagnosed as DM, and nine patients (22%) were diagnosed as IMNM. The muscle strength test “Manual Muscle Testing MMT8” (range 0–80) was lower than normal levels. PCR values were in the normal range, while Cpk was higher than the normal range. For histological and biomolecular analysis, eight DM patients, six IMNM patients, and eight control subjects (CTRL, no-IIM) attending the outpatient clinic at the University of Bari underwent muscle biopsy by open surgery for diagnostic purposes. The muscle samples were immediately frozen in isopentane precooled in liquid nitrogen.

Forty percent of DM patients were positive for anti-Mi-2 autoantibody. Eighty percent of IMNM patients were anti-HMGCR autoantibody positive (Table 1). 

### 2.2. DM and IMNM Patients Showed Reduced Expression of FNDC5 mRNA in Muscle Biopsies

**Figure 1:**
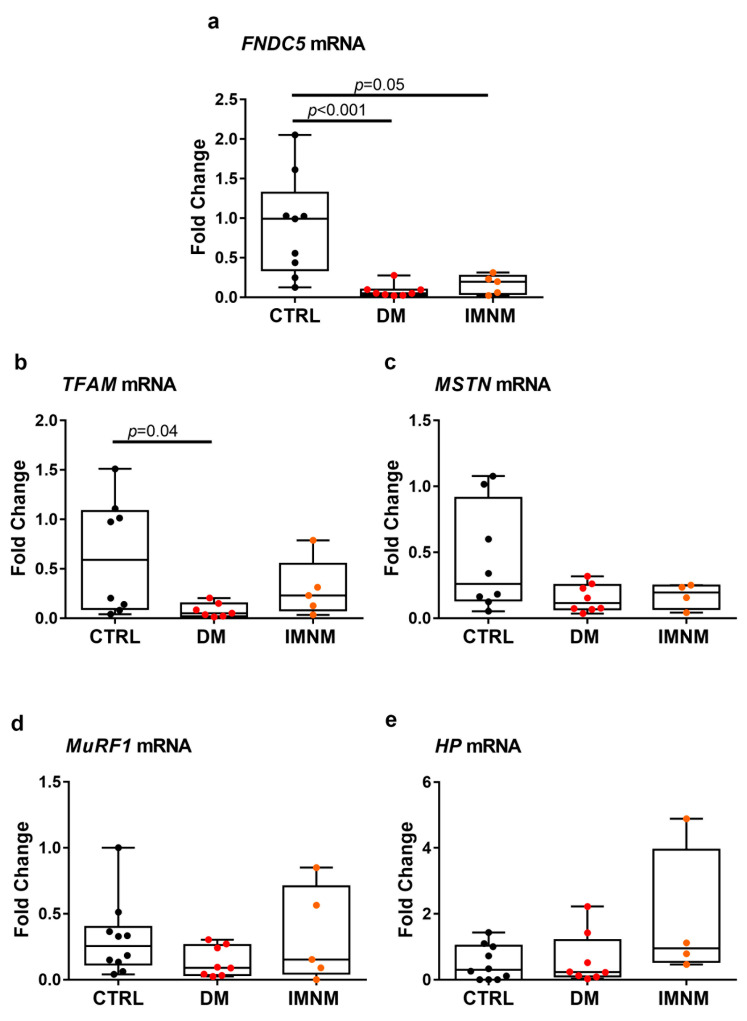
Quantitative PCR (qPCR) showing mRNA expression levels of FNDC5 (**a**), *TFAM* (**b**), *MSTN* (**c**), *MuRF1* (**d**), and *HP* (**e**) in muscle biopsies of control subjects (CTRL) and DM and IMNM patients. Gene expression was normalized to the mean of housekeeping genes (*GAPDH* and *B2M*) and plotted as fold increase from CTRL. D’Agostino and Pearson normality test and Kruskal–Wallis test with two-group comparison by Dunn’s multiple comparisons test were performed. Data are presented as box-and-whisker plot with median and interquartile ranges from max to min with all data points shown; significant results were indicated by *p* value.

Real-Time PCR analysis was performed on muscle biopsies of eight DM patients, six IMNM patients, and eight CTRL subjects among those recruited for this study. Results showed the gene expression of the irisin precursor, FNDC5, was significantly reduced in both DM (*p* = 0.0009) and IMNM (*p* = 0.05) compared with CTRL (Figure 1a). In DM patients, we also found a lower expression of the mitochondrial transcription factor A (*TFAM*) than CRTL (*p* = 0.04), while the difference was not significant in IMNM patients, although a downward trend compared with CTRL can be detected (Figure 1b). We observed no significant differences in the expression of the negative regulator of muscle mass, myostatin (*MSTN)*, in both DM and IMNM compared with controls. Surprisingly, mean MSTN values in both groups of patients tended to be lower than control subjects (Figure 1c). Similarly, no change was detected in the expression of the muscle-specific E3 ubiquitin ligase, *MuRF1* (Figure 1d), and haptoglobin (*HP*) (Figure 1e) mRNA levels. It should be noted, however, the expression of *HP*, a proinflammatory glycoprotein, tended to be higher in patients with IMNM.

### 2.3. Skeletal Muscle Fibers of DM and IMNM Patients Showed Reduced FNDC5 Immunoreactivity 

**Figure 2:**
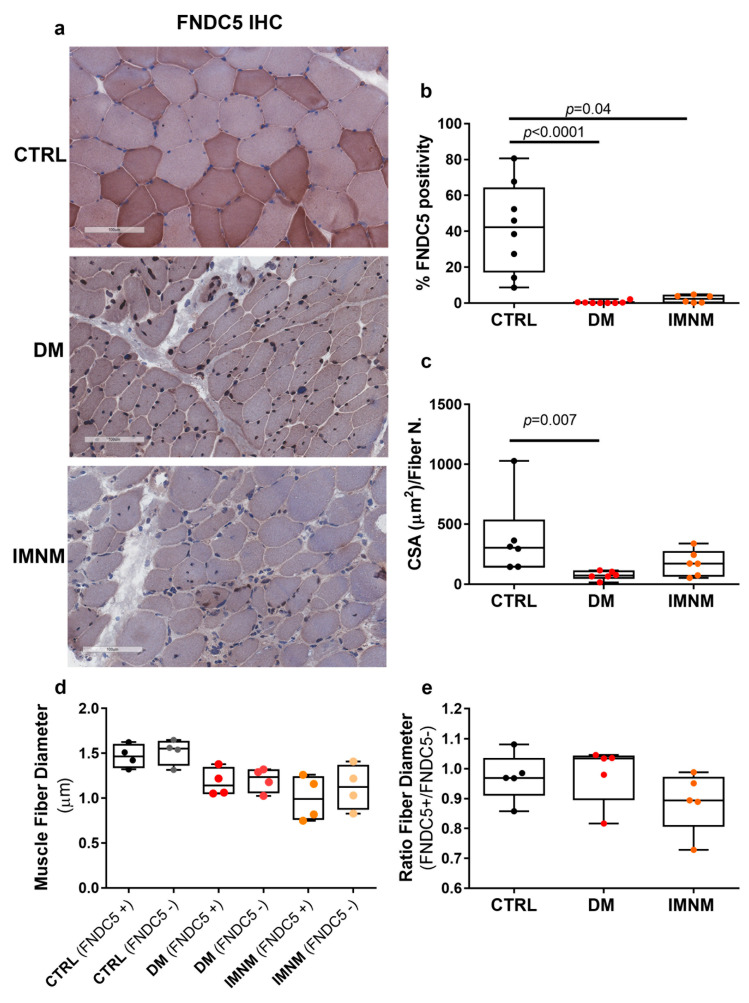
Representative images of immunohistochemistry staining of FNDC5 protein in muscle biopsies of control subjects (CTRL) and DM and IMNM patients (magnification: 20×) (**a**). Quantitative assessment of percentage of FNDC5 staining (**b**). Quantitative assessments of cross-sectional area (CSA) normalized to the number of fibers in muscle biopsies of control subjects (CTRL) and DM and IMNM patients (**c**). Quantitative assessments of diameters of FNDC5(+) and FNDC5(−) fibers in muscle biopsies of control subjects (CTRL) and DM and IMNM patients (**d**) and their ratio (**e**). D’Agostino and Pearson normality test and Kruskal–Wallis test with two-group comparison by Dunn’s multiple comparisons test were performed. Data are presented as box-and-whisker plot with median and interquartile ranges from max to min with all data points shown; significant results were indicated by *p* value.

By immunohistochemistry on OCT sections processed from muscle biopsies, we assessed FNDC5 positivity in DM and IMNM patients. As shown in Figure 2a, in both groups of patients, a strong reduction of FNDC5-positive fibers could be detected compared with muscles from control subjects. Histomorphometry showed the percentage of FNDC5-positive fibers was significantly reduced in both DM (*p* < 0.0001) and IMNM (*p* = 0.04) compared with controls (Figure 2b). In addition, to determine the presence of muscle atrophy, the cross-sectional area (CSA) of muscle fibers normalized to the number of fibers was measured, showing patients with DM had significantly lower CSA (*p* = 0.007), while CSA in patients with IMNM showed a decreasing trend, although not significant, compared with controls (Figure 2c). Therefore, the reduced expression of the transmembrane protein FNDC5 in muscle fibers of these patients suggests the possible implication of its deficiency in muscle atrophy caused by myositis. Moreover, since immunohistochemistry showed FNDC5-positive or negative muscle fibers were clearly distinguishable, particularly in control subjects, we measured their diameters. However, quantization showed there were no significant differences between FNDC5(+) and FNDC5(-) within the same group (d) and thus difference in their ratio (e).

### 2.4. Irisin Levels in Patients with DM and IMNM Are Higher Than in Control Subjects

**Figure 3:**
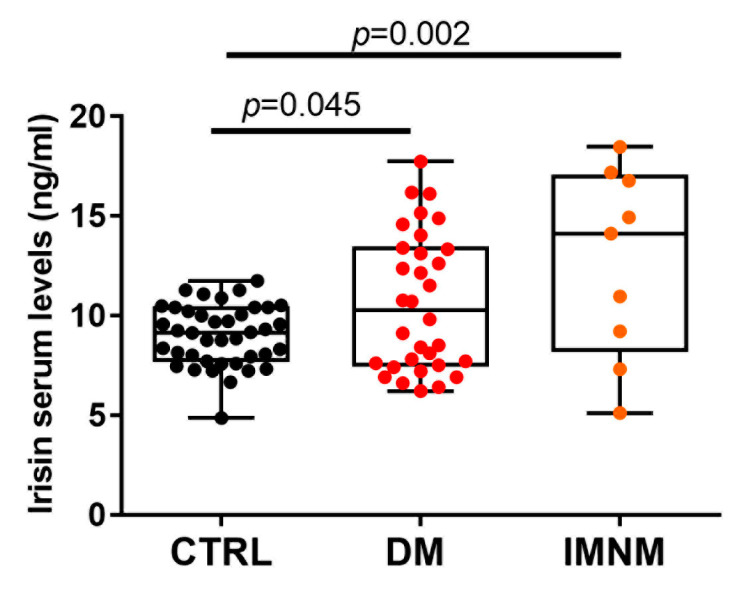
Irisin serum levels (ng/mL) were significantly higher in both DM and IMNM patients compared to control subjects. D’Agostino and Pearson normality test and one-way analysis of variance (ANOVA) with the Tukey’s multiple comparisons test were performed. Data are presented as box-and-whisker plot with median and interquartile ranges from max to min with all data points shown; significant results were indicated by *p* value.

We compared irisin serum levels in DM (N= 32) and IMNM (N= 9) patients with control subjects matched for age and sex (N = 40). Not as expected, circulating levels of irisin were higher in DM (*p* = 0.045) and in IMNM (*p* = 0.002) than in control subjects (Figure 3). This result suggested the imbalance between the high concentration of circulating irisin and the reduced expression of FNDC5 in muscle fibers could depend on abnormal cleavage of its precursor.

### 2.5. Up-Regulated Levels of ADAM10 mRNA in Skeletal Muscles of DM and IMNM Patients

**Figure 4:**
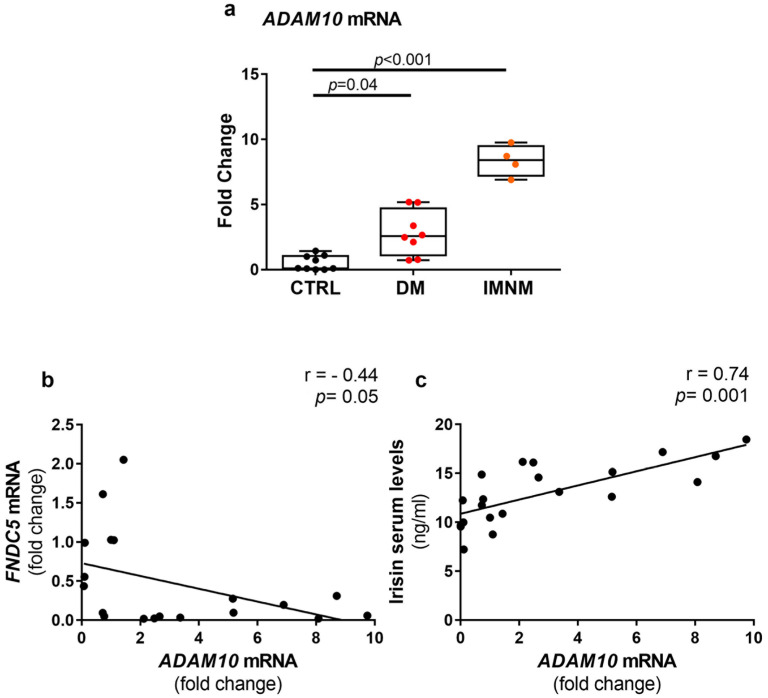
Quantitative PCR (qPCR) showing mRNA expression levels of ADAM10 in muscle biopsies of control subjects (CTRL) and DM and IMNM patients. Gene expression was normalized to the mean of housekeeping genes (*GAPDH* and *B2M*) and plotted as fold increase from CTRL (**a**). D’Agostino and Pearson normality test and Kruskal–Wallis test with two-group comparison by Dunn’s multiple comparisons test were performed. Data are presented as box-and-whisker plot with median and interquartile ranges from max to min with all data points shown; significant results were indicated by *p* value. *FNDC5* mRNA levels negatively correlated with *ADAM10* mRNA levels (**b**.) Irisin serum levels are positively associated with *ADAM10* mRNA levels (**c**). For linear regression, r and *p* values are as indicated.

Given the opposing trends between serum irisin levels and FNDC5, we evaluated the expression of ADAM10, the newly discovered cleavage agent for FNDC5 [26]. Our results showed ADAM10 mRNA levels were significantly higher in skeletal muscle biopsies of DM (*p* = 0.04) and IMNM (*p* < 0.001) patients than in controls (Figure 4a). Notably, we found an inverse correlation between FNDC5 and ADAM10 mRNA fold changes (r = −0.44; *p* = 0.05) (Figure 4b), whereas a stronger positive correlation was observed between irisin serum levels and ADAM10 mRNA fold changes (r = 0.74; *p* = 0.001) (Figure 4c).

## 3. Discussion

There is currently convincing evidence for a link between the myokine, irisin, and its positive effects on skeletal muscle mass. To the best of our knowledge, no studies have been conducted to date that analyzed the expression of the irisin precursor, FNDC5, and circulating levels of irisin in patients with IIM. Our results reveal FNDC5 expression in skeletal muscle of DM and IMNM patients is severely reduced compared with control subjects diagnosed as non-IIM. In agreement with this result, we observed a high degree of muscle loss in DM patients, showing a reduction in muscle CSA comparable to those observed in IMNM in which muscle atrophy and weakness is usually the main clinical manifestation [29]. In support of our observation, a previous study demonstrated Anti-Mi2 positive DM patients showed elevated muscle impairment characterized by necrosis and macrophage infiltrates in myofibers [30]. However, as shown by biomolecular analysis of the biopsies, the reduction in muscle fiber size was not accompanied by significant increase of *MuRF1*, the atrophy marker involved in muscular ubiquitination and protein degradation [31], and *HP*, the haemoglobin-binding protein which is highly produced in response to the acute phase of inflammation [32]. Gene expression levels of *MSTN*, a known negative regulator of muscle mass [33], were also unchanged in DM and IMNM patients, and contrary to expectation, mean of mRNA values in patients were lower, although not significantly, than controls. Interestingly, biomolecular analysis of the biopsies showed reduced levels of *TFAM* in DM and although not significant, also in IMNM patients. The mitochondrial transcription factor, *TFAM*, is a key component of proper mitochondrial DNA function, and numerous studies have shown a direct link between mitochondria dysfunction and skeletal muscle damage [34].

In contrast to the reduced expression of FNDC5, in the present study we observed increased levels of circulating irisin in DM and IMNM patients. In one of our previous studies, we found in muscle biopsies from older adult subjects, FNDC5 immunoreactivity as well as its mRNA levels were consistent with circulating irisin levels. However, the patients recruited in that study were diagnosed as osteoporotic, and no muscle damage was evidenced, such as no modulation of the senescence marker, p21, in skeletal muscle was found [23]. Data achieved in mouse models showed irisin treatment prevents muscle wasting and mitochondrial dysfunction during musculoskeletal unloading [35] and plays a pro-myogenic role by rescuing denervation-induced atrophy [36]. In relation to FNDC5 expression in muscle fibers, we previously observed no change in the number of FNDC5-positive fibers in irisin-treated unloading mice, either compared with vehicle-treated unloading mice or healthy control mice [35]. However, we observed the co-localization of fibers positive for FNDC5 and ATP-synthase was reduced in unloading mice, whereas irisin treatment retained the same co-localization as in healthy control mice, thus indicating a possible alteration in the expression of FNDC5 and muscle fiber type (slow- and fast-twitch) in skeletal muscle in the presence of muscle disease [35]. It is conceivable the marked difference between FNDC5-positive and negative fibers that we observe in the present work could be attributable to an imbalance between fiber type in the patients’ skeletal muscle.

Studies in humans suggested irisin levels may be predictive of sarcopenia in postmenopausal women [37] and of muscle atrophy in patients affected by the inherited polyneuropathy, Charcot-Marie-Tooth [38]. However, to the best of our knowledge, no study in cohorts of subjects with muscle disease investigated irisin serum levels and FNDC5 expression in muscle biopsies at the same time. It is conceivable that in the presence of a myopathy, there may be a discrepancy between circulating irisin and the expression of its precursor in muscle fibers. It must also be considered that in myopathies there could also be a sort of irisin-resistance in the skeletal muscle. Therefore, in the presence of muscle pathology, elevated serum levels of the myokine could be a consequence of its poor utilization.

To decipher the imbalance between irisin and FNDC5, we also analyzed the expression levels of ADAM10, recently shown to be the cleavage agent for FNDC5 in cardiac muscle [26]. Our findings demonstrated the expression of ADAM10 was strongly up-regulated in DM and IMNM patients compared with controls. It is conceivable that in DM and IMNM patients, since there are abnormal levels of ADAM10, the hyper-regulation of FNDC5-cleavage is responsible for an excessive release of irisin into the circulation, thus keeping the expression of its precursor very low in the skeletal muscle. In favor of this hypothesis, we observed a strong positive association between circulating serum irisin and ADAM10 expression in muscle, implying higher circulating levels of myokine observed in DM and IMNM could be caused by up-regulation of FNDC5 cleavage. Consistently, although to a lesser extent, we observe FNDC5 expression is inversely correlated with the expression of its cleavage agent.

There are currently no studies that can support the hypothesis of an over-production of ADAM10 in IIM. One previous study demonstrated the expression of ADAMs correlates with the pathological condition of IIM, and among the ADAM members analyzed, precisely ADAM10 was detected only in skeletal muscle [28]. However, authors reported its expression was independent of inflammatory disease activity, suggesting ADAM10 may have a physiological role rather than pathological within the muscle tissue [28].

Further studies are desirable to ascertain in vitro and in vivo whether FNDC5 is cleaved in an ADAM10-dependent manner in skeletal muscle, as already demonstrated in cardiac muscle [26]. Although the correlations reported in the present study do not provide direct evidence that increased serum irisin levels are caused by increased expression of ADAM10, future studies may corroborate this hypothesis in understanding the hitherto undiscovered physiological mechanism of FNDC5 cleavage in skeletal muscle via ADAM10. Nevertheless, since proteolytic breakdown is critical for immune cell infiltration thus ensuing tissue destruction in muscle inflammatory disorders, new investigations aimed at understanding the role of ADAM10 in idiopathic inflammatory myopathies are needed.

## 4. Materials and Methods

### 4.1. Study Population

We conducted an observational cohort study including forty control subjects (diagnosed as no-IIM) and forty-one patients with IIM (32 DM and 9 IMNM patients) of both sexes recruited from the rheumatology unit (Policlinico of Bari, Italy).

All patients included in the present study fulfilled the 2017 EULAR/ACR classification criteria [39] with a score ≥ 7.5 without biopsy or ≥8.7 with muscle biopsy, corresponding to a probability ≥90% (definite IIM). The presence of myositis-specific and -associated autoantibodies was determined in all patients by commercially available enzyme immunoassay (MYO12D-24, D-Tek, Mons, Belgium) that can detect autoantibodies against Jo-1, PL-7, PL-12, EJ, SRP, Mi-2, MDA-5, TIF1-γ, HMGCR, SSA/Ro52, SAE-1/2, and NXP-2 antigens. Patients with pathognomonic skin manifestations were classified as DM, while patients without skin manifestation but with muscle findings of necrotizing myopathy and/or positive result of autoantibodies characterizing IMNM (anti-HMGCR or anti-SRP) were classified as IMNM [29]. Patients diagnosed with IBM who showed rimmed vacuoles on muscle biopsy or patients diagnosed with other idiopathic inflammatory myopathies were excluded. Patients attending the outpatient clinic at the University of Bari underwent muscle biopsy for diagnostic purposes. Of these, muscle samples from eight DM patients, six IMNM patients, and eight control subjects (CTRL, no-IIM) were available for histological and biomolecular analysis. The muscle samples were obtained by open surgery by a dedicated surgeon and immediately thereafter were frozen in isopentane precooled in liquid nitrogen. Serum irisin levels of CTRL subjects (N = 40) were obtained from age- and sex-matched no-IIM subjects. No study subjects changed their lifestyle at least three months before enrollment. Subjects were asked not to engage in physical activity on the day before the blood and muscle biopsy collections. Patients gave informed consent for muscle biopsy for the diagnostic procedure and for research purposes. Ethical approval was not required for the use of routinely collected anonymized data in this observational study.

### 4.2. Immunohistochemistry

Muscle samples were fixed and cryopreserved in optimal cutting temperature (OCT) compound (VWR Chemicals, Milano, Italy) at −80 °C. Five micrometer (5-μm) sections were collected on Vectabond-treated slides (Vector Laboratories, Burlingame, CA, USA) and subjected to immunoblotting. The following protocol was performed: pre-treatment with acetone/methanol to eliminate OCT; treatment with protease (Proteinase K; Roche, Indianapolis, IN, USA) 0. 1 mg/mL in PBS for 2 min at 37 °C; blocking endogenous alkaline phosphatase in dual endogenous enzyme-blocking target (Agilent Dako, Santa Clara, CA, USA) at room temperature (RT) for 10 min; incubation with rabbit-FNDC5 (diluted 1. 200; Abcam, Cambridge, UK) at RT for 30 min; detection with biotinylated secondary antibody (AB2; Agilent Dako REAL™ Detection System, Santa Clara, CA, USA) at RT for 15 min, streptavidin alkaline phosphatase (Agilent Dako REAL™ Detection System, Santa Clara, CA, USA) at RT for 15 min, chromogen (RED) (Agilent Dako, Santa Clara, CA, USA) at RT for 20 min; counterstaining with Mayer’s hematoxylin (Sigma-Aldrich, St. Louis, MO, USA); mounting in glycergel (Agilent Dako, Santa Clara, CA, USA). For negative controls, the same procedure was followed omitting the primary antibodies. All observations were performed with a Nikon Eclipse 80i light microscope (Nikon Europe B.V., Amstelveen, The Netherlands) at magnification of 20× by using the NIS-Element BR 4.10.00 software.

### 4.3. Real-Time PCR

Muscle biopsies were homogenized with Ultra-Turrax T8 (Ika, Staufen im Breisgau, Germany), and then, total RNA was extracted by RNeasy Mini Kit (Qiagen, Hilden, Germany). Reverse transcription was performed by iScript Reverse Transcription Supermix (Bio-Rad Laboratories, Hercules, CA, USA) in the thermocycler (My cycler; Bio-Rad Laboratories, Hercules, CA, USA). Real-time PCR on the CFX96 real-time system (Bio-Rad Laboratories, Hercules, CA, USA) was performed by SsoFast EvaGreen Supermix (Bio-Rad Laboratories, Hercules, CA, USA) for 40 cycles (denaturation 95 °C for 5 s; annealing/extension 60 °C for 10 s) after an initial 30-s phase for enzyme activation at 95 °C. The primers used were designed with Primer Blast (https://www.ncbi.nlm.nih.gov/tools/primer-blast/, accessed on 26 January 2023). All primers span an exon–exon junction. Sequences of primers are provided in Table 2, where the accession number (NM_) and product length for each primer are also specified. The geometric mean of two housekeeping genes (*GAPDH*, *B2M*) was used to normalize the data. Each transcript was analyzed in triplicate, and quantitative measures were calculated by the ΔΔCT method and expressed as fold change from control.

### 4.4. Determination of Serum Irisin

Serum samples were obtained after centrifugation (1600× *g* at 4 °C for 15 min) and then stored at −80 °C. Serum concentration of irisin was measured by a competitive ELISA kit (Phoenix Pharmaceuticals, Burlingame, CA, USA; Cat. No. EK-067-29), following the manufacturer’s instructions. This kit has sensitivity 1.29 ng/mL and linear range 1.29–27.5 ng/mL, intra-assay coefficient of variation (CV) < 10%, and inter-assay CV < 15%. In this assay, the immunoplate is pre-coated with a secondary antibody, whose non-specific binding sites are blocked. The secondary antibody can bind to the Fc fragment of the primary antibody. This primary antibody’s Fab fragment then binds competitively both the biotinylated peptide and the targeted peptide in the standard curve samples and the unknown samples. The biotinylated peptide interacts with streptavidin-horseradish peroxidase (SA-HRP) which catalyzes the substrate solution. The intensity of the resulting yellow color is inversely proportional to the amount of irisin, measured by spectrophotometer (Eon; BioTek Instruments, Inc., Winooski, VT, USA).

### 4.5. Statistical Analysis

Analysis of sample distribution was performed by D’Agostino and Pearson normality test. Parameters with normal distribution were expressed as mean ± standard deviation (SD), while parameters with non-normal distribution were expressed as median and interquartile range (IQR). By using GraphPad Prism (GraphPad Software, Inc., La Jolla, CA, USA) for values that passed the normality test, we performed one-way analysis of variance (ANOVA) with the Tukey’s multiple comparisons test, whereas for non-normal distributed values, we performed the Kruskal–Wallis test with two-group comparison by Dunn’s multiple comparisons test, comparing the mean rank of each group of patients with the mean rank of the control group. For linear regression analysis, Pearson’s correlation coefficient was used to compare parameters with normal distribution, whereas parameters with non-normal distribution were evaluated with the Spearman’s coefficient. All data are presented as boxplots with medians, interquartile ranges, and maximum and minimum values. Differences were considered significant at *p* < 0.05.

## Figures and Tables

**Table 1:** Demographic, anthropometric, skeletal muscle mass, and laboratory parameters of patients. Mean (±SD) or median (25%; 75%) for all variables.

	CTRL (N = 40)	DM (N = 32)	IMNM (N = 9)
Age (years)	58.03 ± 17.98	57.09 ± 19.25	57.78 ± 12.98
Sex (Number)	8 M; 33 F	6 M; 26 F	2 M; 7 F
Irisin (ng/mL)	8.99 ± 1.51	10.25 (7.52; 13.37)	12.66 ± 4.73
MMT8 Unilaterally (potential score 0–80)	n/a	73 (66; 79)	65.11 ± 9.47
Cpk (60–190 U/L)	n/a	395 (76; 1559)	3327 (1155; 4068)
PCR (>5–10 mg/L)	n/a	6.22 ± 4.76	7.94 ± 4.25
Auto-antibodies	n/a	Mi-2 (40%DM) MDA-5 (19%DM) TIF1-γ (15.6%DM) NXP-2 (9.4%DM) SAE-1/2 (9.4% DM) SSA/Ro52 (3.1%DM)	HMGCR (80%IMNM)SRP (11%IMNM)

**Table 2:** Table sequence, accession number (NM_), and product length for each primer.

Gene Name and ID	Forward (5′–3′)	Reverse (5′–3′)	Product Length
*GAPDH* NM_001256799.3	aatgggcagccgttaggaaa	gcccaatacgaccaaatcagag	166
*B2M* NM_004048.4	agatgagtatgcctgccgtg	ttcaaacctccatgatgctgc	97
*FNDC5* NM_001171940.2	tcatcgtcgtggtcctgttc	tcaatgatgtcatactggcggc	70
*MSTN* NM_005259.3	caggcactggtatttggcag	aacggattcagcccatcttctc	163
*HP* NM_001126102.3	cgccacagaaggagatggag	ttgggcttcccacatactgc	108
*MuRF1* NM_032588.4	gagccaccttcctcttgactg	ctcagggcgtctgctatgtg	145
*ADAM10* NM_001110.4	tcatggtgaaacgcataagaatca	ccagaccaagtacgccatca	183
*TFAM* NM_001270782.2	cgggttccagttgtgattgc	acacaaaactgaagggggagc	196

## Data Availability

The data that support the findings of this study are available on request from the corresponding author.
